# Near-miss hypoglycemia—reflections on perioperative glucose management guidelines in diabetics

**DOI:** 10.1186/s12871-023-02145-6

**Published:** 2023-06-01

**Authors:** Rabeel Ahmad, Rotem Naftalovich, George Tewfik, Jean Daniel Eloy, Daniel T. Rodriguez-Correa

**Affiliations:** 1 Department of Anesthesia & Perioperative Care, Rutgers - New Jersey Medical School, Newark, NJ USA; 2 Department of Anesthesia & Perioperative Care, Rowan University School of Osteopathic Medicine, Stratford, NJ USA; 3 Medical Corps of the U.S. Army, U.S. Army Medical Department, Fort Sam Houston, San Antonio, TX USA

**Keywords:** Diabetes, Anesthesia, Perioperative, Blood glucose, Insulin

## Abstract

**Background:**

The American Society of Anesthesiologists (ASA) has an impressive array of professional perioperative guidelines but has not issued a guideline specific to perioperative blood glucose management and does not delve into the topic in their other guidelines.

**Case report:**

We experienced a perioperative case that highlights the potential difficulty of glucose management in this setting. During anesthetic induction for an orthopedic foot surgery, as the medication was infusing, an IDDM 1 (insulin dependent diabetes mellitus type 1) patient expressed feeling that her blood sugar level was low. Her finger stick after induction showed severe hypoglycemia with a blood glucose of 34 mg/dL. The hypoglycemia was treated with intravenous glucose and further closely monitored.

**Conclusions:**

This case led us to revisit the different perioperative guidelines and recommendations for diabetic patients and this manuscript aims to highlight the similarities and discrepancies among the different published recommendations. This case highlights the value of utilizing insulin pump infusions in the perioperative setting when available.

## Background

Diabetes mellitus is a common chronic disorder affecting approximately 10% of the United States population. The dependence of the brain on glucose as its main energy source makes it susceptible to hypoglycemia. An awake patient is capable of sensing hypoglycemia—a particularly meaningful form of monitoring in diabetics taking insulin (both type 1 and type 2). If hypoglycemia is not treated, under general anesthesia, mental status changes (e.g. lightheadedness, headache, or confusion) will be masked and if paralysis is used, as is often the case, convulsions will not be observed. If undetected, hypoglycemia can lead to undesirable consequences [10]. Even though general anesthesia reduces glucose utilization by dramatically reducing cerebral metabolic rate, serious attention should be given to a patient complaining of feeling hypoglycemic, particularly in a type 1 DM patient.

In type 1 DM pancreatic β-cells are destroyed and therefore endogenous insulin cannot be secreted [8]. This means that the physiology of type 1 DM patients is completely dependent on exogenous insulin [11]. The inability to secrete endogenous insulin in type 1 DM can result in a neuroendocrine stress response with hypersecretion of catabolic hormones during surgery or anesthesia. In contrast, Type 2 diabetes is characterized by hyperglycemia, insulin resistance, and also relative impairment in insulin secretion [13]. The strong clinical association of type 2 DM with obesity is supported by the presence of insulin resistance. However, the current classification of diabetes into Type 1 and Type 2 may be overly simplistic for capturing the varied presentations, disease course, medication response, or complications of patients with different genetic phenotypes [6]. The American Society of Anesthesiologists (ASA) has not issued a guideline specific to perioperative blood glucose management in any patient group, in particular, in patients with any form of diabetes mellitus. The existing guidelines that address perioperative blood glucose management are pertinent but the variability among them complicates their clinical applicability. This manuscript aims to highlight the similarities and discrepancies among the different published recommendations.

## Case report

A 36 year old female with hypothyroidism (well controlled with levothyroxine), insulin dependent diabetes mellitus 1 (IDDM 1), and a BMI of 18 presented for an elective open reduction and internal fixation (ORIF) procedure of a closed right trimalleolar fracture. She used an insulin pump for blood glucose management and discontinued her pump insulin infusions at 10 pm on the night before surgery after which she remained NPO for the procedure. Of note, her HbA1c was 10.6% a month before her procedure.

**Fig. 1:**
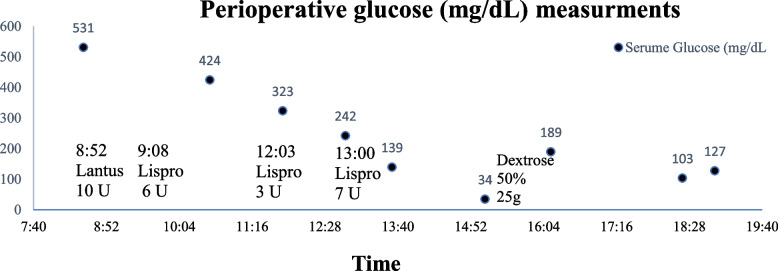
The patient’s glucose readings throughout her procedure day along with the corresponding treatments

On arrival, she presented hyperglycemic with a fingerstick glucose of 531 mg/dL. Her potassium on arrival was 5.2 mmol/L. The pre-operative treatment of her hyperglycemia on the morning of the surgery is shown in Fig. 1; she received insulin glargine (Lantus) 10 units (8:52 am) and a total of 16 units of insulin human lispro (Humalog) subcutaneously; 6 units (9:08 am), 3 units (12:03 pm), 7 units (1:00 pm). Prior to the surgery glucose measurement, by point-of-care finger stick, was 139 mg/dL (1:35 pm) and a decision was made to hold any further hyperglycemia treatment and proceed with surgery. A regional nerve block as a primary anesthetic was not chosen for surgical preference. Surgery started at 3pm with anesthesia induction using 100 mcg of fentanyl, 2 mg of midazolam, 200 mg of propofol, and 50 mg of rocuronium. During anesthetic induction, as the medications were being administered, the patient complained of feeling that her blood sugar was low. Her verbal complaint was the impetus to repeat a finger-stick glucose measurement immediately after induction revealing hypoglycemia of 34 mg/dL which was treated with intravenous 25 g of Dextrose 50%. Her next potassium measurement was 4.0 mmol/L at 11:05 pm.

## Discussion and conclusions

Obtaining a pre-operative glucose measurement prior to induction of general anesthesia is prudent in any diabetic patient. But the timing of this pre-operative glucose check is less clear-cut as it involves judgment based on multiple parameters such as recent prior glucose values, the types of medications given preoperatively for glycemic control and their timings, as well as NPO status. In this particular case our last glucose check was 0.5 h after administration of regular insulin or 1.5 h prior to anesthesia induction. Regular insulin has an onset of action of about 30 min and its duration peaks between 2 and 4 h after dosing. The patient received long-acting insulin glargine (Lantus) 6 h prior to surgery; though it does not really peak, its duration of action can be as long as 12–24 h. This, combined with the regular insulin, whose effect was still increasing at the time of induction, meant that the last pre-op check at 1.5 h prior to induction did not capture the nadir glucose trajectory. In hindsight, a pre-operative glucose measurement closer to induction would have been more reflective of the situation.

Sometime patients are scheduled to undergo anesthesia for an elective surgery at a given time but unforeseen developments delay the procedure by a few hours. In this case the hyperglycemia took hours to correct and this resulted in a morning scheduled surgery instead delayed to the afternoon. The unintended consequence of this was an extended NPO period which further contributed to poor glycemic control. Early-on in management, when the hyperglycemia was being aggressively corrected, a low-dose concurring infusion of D5W sugar could have benefited this patient by reducing the likelihood of hypoglycemia from over correction.

If we knew that the patient was hypoglycemic, we would have delayed induction and instead treated the hypoglycemia. However, the patient only started complaining of hypoglycemic symptoms on induction after we started infusing the anesthetic. The intra-operative glucose check immediately after induction was prompted because of inkling from what the patient verbally expressed on induction.

More diabetic patients are using insulin pumps to administer subcutaneous insulin instead of manual subcutaneous injections [1]. Insulin pumps require continuous self-management, including changing infusion sites every two days and manually adjusting insulin levels. These pumps administer insulin continuously via the subcutaneous route through two types of doses: basal (background insulin) and bolus (for increase in blood sugar from meals) insulin. The pumps give insulin based on pre-programmed basal and bolus rates set by the patient and their healthcare provider. Patients are advised to check their glucose levels between 5 and 10 times per day [1].

Perioperatively, patients using an insulin pump may continue with their usual basal infusion rate if the catheter and pump remain in place and if the patient can resume self-management postoperatively [8]. If the pump is to be discontinued at home prior to surgery, basal insulin can be administered two to three hours prior to pump discontinuation [8]. A treatment approach that may have worked better at managing the preoperative hyperglycemia in this patient is to restart her pump insulin infusion. Even if a decision is made to hold pump infusions, it can be beneficial to keep the device on in the perioperative setting and utilize its glucose measurement capabilities. Insulin pumps measure glucose from the interstitium, a distinct physiologic compartment than the blood, and accordingly the readings from these devices are different from those obtained by a finger-stick (capillary glucose) or a venous sample. This difference should be kept in mind when correcting glucose abnormalities perioperatively as the interstitial glucose is in slow equilibrium with capillary blood and therefore pump glucose readings will likely lag behind blood glucose readings [5].

The “Standard of Medical Care in Diabetes” from the American Diabetes Association (ADA), updated in 2021, provides the following seven current clinical practice recommendations and guidelines:

1. The target range for blood glucose in the perioperative period should be 80–180 mg/dL (4.4–10.0 mmol/L).
2.  A preoperative risk assessment should be performed for patients with diabetes who are at high risk for ischemic heart disease and those with autonomic neuropathy or renal failure.
3. Metformin should be withheld on the day of surgery.
4. SGLT2 inhibitors must be discontinued 3–4 days before surgery.
5. Withhold any other oral glucose lowering agents the morning of surgery or procedure and give half of NPH dose or 75–80% doses of long acting analog or pump basal insulin.
6. Monitor blood glucose at least every 2–4 h while patient is taking nothing by mouth and dose with short- or rapid-acting insulin as needed.
7. There are no data on the use and/or influence of glucagon-like peptide 1 receptor agonists or ultra-long-acting insulin analogs upon glycemia in perioperative care [1].

The pump basal infusion of our patient could have been reduced to 75% of baseline as per the 5th ADA guideline instead of discontinued. This would have attenuated the hyperglycemia and maybe also the hypoglycemia from over correction with insulin. It is important to remember that guidelines are just recommendations and that case specific circumstances are paramount; as in our case, a repeat glucose measurement after 1.5 h was not frequent enough for that particular set of circumstances despite the 6th ADA guideline timeline of 2-4 h.

Medical societies such as the Endocrine Society and Association of Anesthetists of Great Britain and Ireland (AAGBI) are mostly in agreement with the above seven guidelines, and so are the major anesthesia textbooks such as *Miller’s Anesthesia* and *Morgan and Mikhail’s Clinical Anesthesiology*. However, there are some noteworthy discrepancies among the different society recommendations. The areas of discrepancy and corroboration between the ADA and other medical societies and clinical anesthesia textbooks are outlined in the table below (Table 1).

**Table 1:** Discrepancies and corroborations between the ADA and other medical societies and clinical anesthesia textbooks. Similarities marked by check mark and discrepancies marked by ‘X’, see text for further details

American Diabetes Association (ADA) *Standard of Care*	Anesthetists of Great Britain and Ireland (AAGBI)	*Miller’s Anesthesia*	*Morgan and Mikhail’s Clinical Anesthesiology*	*Diabetic Perioperative Management*, Dogra & Jialal
1. Target range for blood glucose in the perioperative period should be 80–180 mg/dL			✔	
2. A preoperative risk assessment should be performed for patients with diabetes due to high risk for ischemic heart disease and those with autonomic neuropathy or renal failure				
3. Metformin should be withheld on the day of surgery	**X**	✔	✔	
4. SGLT2 inhibitors must be discontinued 3–4 days before surgery				
5. Withhold any other oral glucose lowering agents the morning of surgery or procedure and give half of NPH dose or 75–80% doses of long acting analog or pump basal insulin	**X**	✔	**X**	
6. Monitor blood glucose at least every 2–4 h while patient is NPO and dose with short- or rapid-acting insulin as needed			**X**	✔
7. There are no data on the use and/or influence of glucagon-like peptide 1 receptor agonists or ultra-long-acting insulin analogs upon glycemia in perioperative care				

Preoperatively, the 3rd and 5th ADA guidelines conflict with the AAGBI recommendations. The AAGBI’s position is that “The common exercise is to hold all oral anti-diabetic medications on the day of undertaking surgery; however, this strategy does not deem fit for those patients undergoing surgery for a shorter period or are expected to resume their diet quickly or are discharged after having a short stay” [13]. Instead, they recommend an “individualized approach with the option of carrying on with the antihyperglycemic medications that do not cause low blood sugars with metformin not considered an exception” [3].

In contrast, both *Morgan and Mikhail* and *Miller* are in agreement with the 3rd and 5th ADA perioperative guidelines. As per *Miller*, “for all patients, discontinue all short-acting (e.g., regular) insulin on the day of surgery (unless insulin is administered by continuous pump)” [9]. Also, *Miller* discusses that “normal treatment regimen for most noninsulin diabetic medications (metformin, sulfonylureas, repaglinide, GLP-1 agonists, DPP-4 inhibitors) should be continued until (and inclusive of) the day before surgery but held on the morning of surgery” [9]. According to *Morgan and Mikhail*, “If the patient is taking an oral hypoglycemic agent preoperatively rather than insulin, the drug can be continued until the day of surgery. However, sulfonylureas and metformin have long half-lives and many clinicians will discontinue them 24 to 48 h before surgery. They can be started postoperatively when the patient resumes oral intake. Metformin is restarted if renal and hepatic function remain adequate” [4].

*Morgan and Mikhail* are also in agreement with the 1st ADA perioperative guideline and note that “The goal of perioperative blood glucose management is to avoid hypoglycemia while maintaining blood glucose below 180 mg/dL” [4].

The 5th ADA perioperative guideline is inconsistent with *Morgan and Mikhail*. The textbook does not support the ADA’s approach of giving half of NPH dose or 75–80% doses of long-acting analog or pump basal insulin because it is not “terribly effective” [4]. Butterworth et al. recommend administering regular insulin intraoperatively as a continuous infusion in all but short procedures [4]. They argue a continuous infusion has more precise control of insulin delivery than a subcutaneous or intramuscular injection of NPH insulin, particularly in conditions associated with poor skin and muscle perfusion [4].

Because anesthesia masks the signs and symptoms of hypoglycemia, blood glucose monitoring is generally more frequent in the perioperative setting, particularly in diabetic patients undergoing general anesthesia. A reasonable approach is to tailor intra-operative glucose monitoring based on the length and type of surgery. Accordingly, some clinicians suggested that “surgeries of shorter duration (<less than 4 hours) with expected hemodynamic stability and minimal fluid shift, can be managed with 2-hourly subcutaneous correctional insulin (preferably rapid-acting insulin) and BG [blood glucose] checks” [7]. On the other hand, “surgeries that may involve hemodynamic fluctuations, massive fluid shifts, or last longer than 4 hours duration, BG greater than 180 mg/dL should be managed with intravenous insulin, and BG should monitored every 1 to 2 hours” [7]. This approach is in overall consensus with the ADA’s 6th guideline, suggesting monitoring blood glucose every 2–4 h [1].

*Morgan and Mikhail* contrast by arguing for a more aggressive blood glucose monitoring approach and note that “The key to any diabetic management regimen is to monitor plasma glucose levels frequently. Patients receiving insulin infusions intraoperatively may need to have their glucose measured hourly” [4].

Regarding post-operative glucose management, *Morgan and Mikhail* notes that “Close monitoring of blood glucose must continue postoperatively. There is considerable patient-to-patient variation in onset and duration of action of insulin preparations. For example, the onset of action of subcutaneous regular insulin is less than 1 h, but in rare patients its duration of action may continue for 6 h. NPH insulin typically has an onset of action within 2 h, but the action can last longer than 24 h” [4]. The 2021 ADA guidelines and *Miller* do not offer any specific postoperative glucose management approaches.

Type 1 diabetics are completely dependent on the administration of exogenous insulin, fluid therapy, and glucose to prevent diabetic ketoacidosis. As a result, diabetic ketoacidosis is a particular concern in type 1 diabetics. Concurrent administration of insulin, glucose and potassium should be considered perioperatively until oral intake is resumed. When possible, intravenous glucose infusion and insulin therapy should be continued when a Type 1 diabetic is NPO. Recent NPO guidelines that allow clear liquids to be consumed up to two hours prior to surgery can assist preoperatively in avoiding and treating hypoglycemia.

Perioperative blood glucose management is not currently addressed by the ASA guidelines. Different approaches and recommendations exist. Regardless of the clinician’s preference, vigilance is paramount in perioperative glucose management and patient specific judgment is particularly valuable in glucose management decisions

## Data Availability

Provided in Works Cited.

## Abbreviations

IDDM 1: Insulin Dependent Diabetes Mellitus 1
DM: Diabetes Mellitus
